# COVID-19 and cardiovascular disease in patients with chronic kidney disease

**DOI:** 10.1093/ndt/gfad170

**Published:** 2023-09-28

**Authors:** Lucia Del Vecchio, Olga Balafa, Evangelia Dounousi, Robert Ekart, Beatriz Fernandez Fernandez, Patrick B Mark, Pantelis Sarafidis, Jose M Valdivielso, Charles J Ferro, Francesca Mallamaci

**Affiliations:** Department of Nephrology and Dialysis, Sant'Anna Hospital, ASST Lariana, Como, Italy; Department of Nephrology, University Hospital of Ioannina, Ioannina, Greece; Department of Nephrology, Faculty of Medicine, School of Health Sciences, University of Ioannina, Ioannina, Greece; Department of Dialysis, Clinic for Internal Medicine, University Medical Center Maribor, Maribor, Slovenia; Department of Nephrology and Hypertension, IIS-Fundacion Jimenez Diaz UAM, Madrid, Spain; School of Cardiovascular and Metabolic Health, University of Glasgow, Glasgow, UK; 1st Department of Nephrology, Hippokration Hospital, Aristotle University of Thessaloniki, Thessaloniki, Greece; Vascular and Renal Translational Research Group, Institute for Biomedical Research on Lleida (IRBLleida), Lleida, Spain; Department of Renal Medicine, University Hospitals Birmingham and Institute of Cardiovascular Sciences, University of Birmingham, Birmingham, UK; Francesca Mallamaci Department of Nephrology, Dialysis, and Transplantation Azienda Ospedaliera “Bianchi-Melacrino-Morelli” & CNR-IFC, Reggio Calabria, Italy

**Keywords:** cardiovascular disease, chronic kidney disease, COVID-19, post-COVID syndrome, SARS-Cov2

## Abstract

Millions of people worldwide have chronic kidney disease (CKD). Affected patients are at high risk for cardiovascular (CV) disease for several reasons. Among various comorbidities, CKD is associated with the more severe forms of severe acute respiratory syndrome coronavirus 2 (SARS-CoV-2) infection. This is particularly true for patients receiving dialysis or for kidney recipients. From the start of the SARS-CoV-2 pandemic, several CV complications have been observed in affected subjects, spanning acute inflammatory manifestations, CV events, thrombotic episodes and arrythmias. Several pathogenetic mechanisms have been hypothesized, including direct cytopathic viral effects on the myocardium, endothelial damage and hypercoagulability. This spectrum of disease can occur during the acute phase of the infection, but also months after recovery. This review is focussed on the CV complications of coronavirus disease 2019 (COVID-19) with particular interest in their implications for the CKD population.


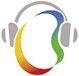
 **Watch the video of this contribution at** https://academic.oup.com/ndt/pages/author_videos

## INTRODUCTION

The coronavirus disease 2019 (COVID-19) [severe acute respiratory syndrome coronavirus 2 (SARS-CoV-2)] pandemic began in late December 2019 in China and became a global, often deadly, disease within months. Many risk factors have been associated with the more severe forms of SARS-CoV-2 infection, with older age and male gender being the most important for increased risk of mortality. In addition to age and male gender, many underlying comorbidities have been found to be associated with mortality of SARS-CoV-2, such as obesity, hypertension, diabetes mellitus,

chronic disease and cancer [1]. Among them, chronic kidney disease (CKD) has a prominent role with a significantly higher risk of mortality than any other category of patients [2]. This serious and peculiar aspect of CKD and the SARS-CoV-2 pandemic has stimulated nephrologists from all over Europe to build up a large European database (ERACODA, the database of the European Renal Association COVID-19) with the aim of investigating the course and outcome of COVID-19 in patients treated with kidney replacement therapy, i.e. living with either a kidney transplant or on maintenance dialysis therapy [3]. Even CKD patients not on dialysis have been reported to have an increased risk of developing serious complications of COVID-19 infection, including acute respiratory distress syndrome (ARDS) or a worsening of kidney function. Among various pre-disposing factors, people with CKD may have weaker immune systems and other underlying health conditions, such as hypertension and diabetes which are known to contribute to an increased risk of serious illness from COVID-19.

Recent studies have shown that COVID-19 infection can lead to a higher risk of cardiovascular (CV) complications in people with CKD potentially because COVID-19 can cause further damage to the heart and blood vessels, in patients already known to have a higher risk of CV diseases. The most common CV complications that have been reported in COVID-19 patients with CKD include myocarditis, pericarditis, thromboembolic events, acute coronary syndrome events and hypertensive emergencies.

This review is intended to cover the spectrum of COVID-19 in CKD patients focussing on CV complications and implications for vaccinations, and possible treatments and prevention strategies in these high-risk patients.

## EPIDEMIOLOGY OF COVID-19 IN THE CKD POPULATION

The SARS-CoV-2 pandemic has almost hit every corner of the world and infected millions of people (as of 10 March 2023, 676 609 955 infections were reported worldwide with 6 881 955 deaths) [4]. Given the high prevalence of CKD in the world population, it is not surprising that thousands of CKD patients got the infection, often with serious consequences. Moreover, especially in the first months of the pandemic, haemodialysis patients could not really isolate since they needed to attend the hospital two to three times a week for the dialysis sessions. Unfortunately, extensive and systematic data on the epidemiology of the infection *per se* in the CKD population are not available. The International Severe Acute Respiratory and Emerging Infection Consortium (ISARIC) COVID-19 is one of the largest international databases of people hospitalized with COVID-19 [5]. As of September 2021, it included more than 700 000 patients hospitalized for COVID-19 worldwide; only 6% of this patient population had CKD [5]. However, these data likely suffer from underreporting.

Since the very beginning of the pandemic, it was evident that CKD was a risk factor for severe COVID-19 [1, 6]. This was further confirmed by larger data sets [6]. The National Health Service of England collected information for 40% of the population in the analytic platform OpenSAFELY [1]; among comorbidities, reduced kidney function was a significant risk factor of death. According to a metanalysis of 84 studies published from May 2020 to January 2021, the presence of CKD was associated with increased mortality [meta-odds ratio 2.13, 95% confidence interval (CI) 1.69–2.67] [6]. Interestingly, the survival probability in CKD patients was influenced by the geographical origin, since population studies based in North American and European were more likely to have a lower estimate for CKD mortality risk than those performed in Asia, South America and Africa [6]. The presence of CKD was also associated with increased need for hospitalization [6].

The risk of developing severe COVID-19 progressively increases with the worsening of CKD [1], with those on dialysis having the highest risk [hazard ratio (HR) 3.69, 95% CI 3.09–4.39] [7]. Kidney transplant recipients are also at high risk of death, especially in the first year after transplantation [7].

The epidemiological evaluation of the role of CKD as a risk factor for severe COVID-19 infection and increased mortality is made complex by the fact that in many instances patients have more than one comorbidity, with many subjects having not only CKD but also obesity, diabetes, hypertension and pre-existing CV disease (CVD). Because of their high burden of comorbidities and/or advanced age, patients with advanced CKD are often not qualified for intensive care and mechanical ventilation, further reducing their probability of survival in case of severe respiratory insufficiency. However, the outcome is poor also for those entering the intensive care unit (ICU), with only 1 in 10 surviving [8].

The discovery and implementation of vaccines against SARS-CoV-2, together with subsequent viral modifications, have contributed to significantly improve outcomes in the general population and in CKD patients as well [9]. However, stage 5 CKD patients remained at high risk despite having received an initial booster [10].

## COVID-19 AND CVD: POTENTIAL MECHANISMS

**Figure 1:**
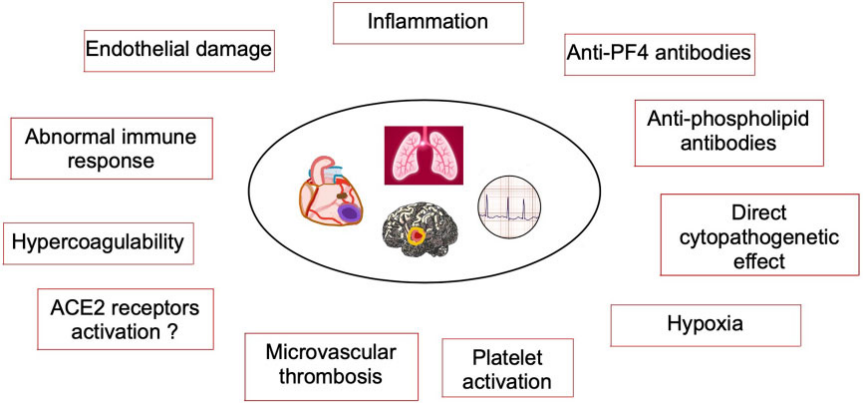
Possible pathogenetic mechanisms of CV involvement of SARS-CoV-2 infection. SARS-Cov2 infection can cause CV manifestations through several mechanisms, including accelerated inflammation, hypocoagulability, microvascular thrombosis, endothelial damage and platelet activation. The role of ACE2 receptor activation and of a direct cytopathogenic effect is still controversial.

CV damage in the setting of SARS-CoV-2 infection has a complex pathogenesis. Beyond the detrimental effects of hypoxemia in the setting of ARDS on the myocardium and decompensation of already established CVD, additional intertwining mechanisms might be implicated during the COVID-19 disease course. Direct cytopathic viral effects on the myocardium, endothelial damage, hypercoagulability and erratic systemic immune responses have been at the centre of attention as the main culprit mechanisms (Fig. 1) [11, 12]. Although the presence of viral replication and coronavirus particles in cardiac tissue obtained at autopsy was occasionally reported early in the pandemic, evidence of direct viral cytopathic effects remains controversial and relies mainly on experimental data [13, 14]. *In vitro* models of human pluripotent stem cell–derived cardiomyocytes indicate that SARS-CoV-2 spread within the myocardial cells occurs via the interaction of activated SARS-CoV-2 spike protein and the angiotensin-converting enzyme 2 (ACE2) receptor, which is highly expressed by human cardiomyocytes [13, 14]. Following assembly into intracellular lysosome-like vesicles, viral transcripts are integrated with cellular mRNA and disrupt cellular metabolism and the genomic machinery, and induce cardiomyocyte fusion and eventually cell death [13, 14]. However, accumulating evidence from autopsy series of patients who died of COVID-19 do not reveal histological stigmata of overt myocarditis except for local inflammatory infiltrates of macrophages and T cells [15, 16]. Instead, microvascular thrombosis and ensuing myocardial cell death appear to be the most prominent findings, which are ascribed mainly to endothelial damage and the pro-thrombotic state [17, 18]. Thus, following direct endothelial cell invasion through the ACE2 receptor, the S protein may induce mitochondrial dysfunction, impaired nitric oxide synthase (eNOS) activity, and downregulation of ACE2 [19]. In addition, platelet hyperreactivity might occur due to direct platelet activation by the spike protein. According to recent evidence, the spike protein might activate the TMEM16F protein in platelets, a calcium-dependent scramblase, which promotes externalization of phosphatidylserine onto the outer leaflet of the plasma membrane, thus enhancing the procoagulant activity of platelets [20]. The hyperinflammatory milieu triggered by SARS-CoV-2 manifests as an imbalance of T-cell activation and exaggerated expression and production of T helper 1 and 2 cytokines such as interleukin 6 (IL)-6 and IL-17, which induce chemotaxis of neutrophil and macrophages, formation of neutrophil extracellular traps (NETs) and inflammasome activation resulting in immune-mediated cardiac injury [11, 12, 21, 22]. A vicious circle ensues with the inflammatory response begetting further endothelial damage, activation of the coagulation cascade, NOX2 upregulation and reactive oxygen species production. The hypothesis that ACE2 receptors located in the CV system might affect the renin–angiotensin–aldosterone system (RAAS) locally leading to cardiomyocyte damage and cardiac remodelling remains speculative at present.

## COVID-19 AND RAAS INHIBITION: IMPLICATION FOR CKD

Shortly after the discovery of the use of ACE2 as the point of entry of the virus in the cells, two contradictory hypotheses regarding the use of RAAS inhibitors (RAASi) in COVID-19 patients emerged. Some authors warned against the potential deleterious effect of RAASi, which has been shown to increase ACE2 expression [23], whereas others argued that the treatment may be beneficial, as it would increase the anti-inflammatory and anti-fibrotic actions of angiotensin 1–7 [24]. Observational studies showed controversial results, ranging from no effect on COVID-19 infection susceptibility [25], or severity of the disease and mortality [26], to improvements in survival in individuals treated with RAASi [27, 28], with beneficial effects observed only in hypertensive patients [29] or only in males [30].

Overall, randomized clinical trials have shown that discontinuation of RAASi has no effect on COVID-19 mortality [31, 32]. However, the BRACE-CORONA trial showed a slight advantage in continuing RAASi treatment in the median length of the hospitalization [32]. On the contrary, the ACEi-COVID trial, which recruited patients older than 80 years, showed that treatment discontinuation led to faster recovery with no effect on the severity of the disease [33]. Clinical trials introducing RAASi *de novo* have been also performed, with mixed results [34, 35]. New clinical trials are underway but, as on many other occasions, CKD patients have been excluded [36, 37]. The only information specific for CKD patients comes from a study by Soler *et al.* [38]. The authors investigated the effects of RAASi on mortality and hospitalization in patients on renal replacement therapy in the large database of ERACODA (see above). The results showed similarities to the general population, as no differences in any of the endpoints between treated and untreated patients were reported. Several questions remain to be answered. Is there a difference between ACE inhibitors and angiotensin receptor blockers (ARB)? Will *de novo* addition of RAASi improve the course of the disease? Only new randomized trials can answer these questions. However, their feasibility depends on whether new recrudescence of the SARS-CoV-2 pandemic will occur in the future.

## COVID-19 AND ACUTE CVD: CLINICAL MANIFESTATIONS

### General population

**Figure 2:**
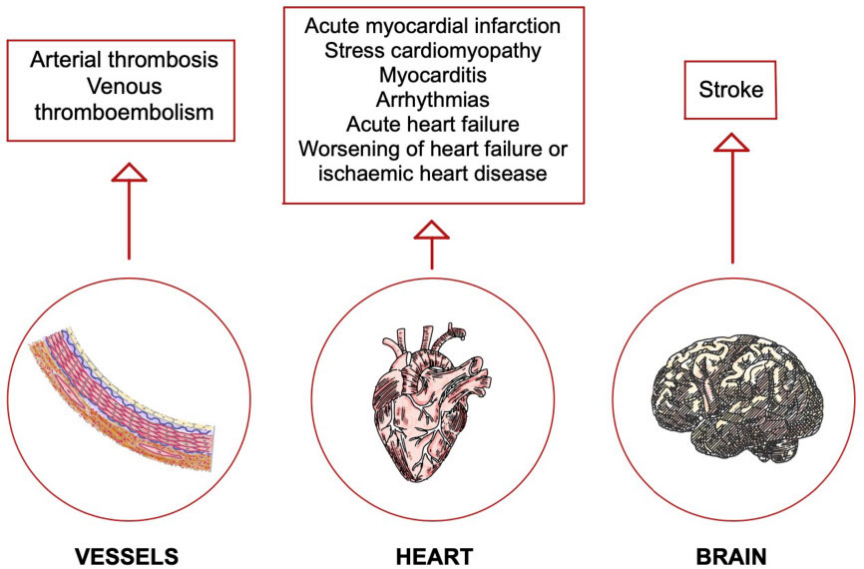
CV involvement of SARS-CoV-2 infection. The SARS-CoV-2 virus can damage the CV system in several ways, from a direct inflammatory effect on the myocardium, leading to myocarditis, to arterial or venous thrombosis occurring in several vascular district. Arrythmias and acute heart failure also develop at increased rate in infected patients.

Preexisting CVD (coronary artery disease, heart failure, cerebrovascular disease), CV risk factors (e.g. male sex, older age, hypertension, diabetes) and other comorbidities (e.g. chronic obstructive pulmonary disease, CKD and cancer) predispose patients with COVID-19 to a more severe disease and higher mortality [39]. COVID-19 may also exacerbate the underlying CVD and cause *de novo* cardiac complications [40] (Fig. 2).

In hospitalized patients with COVID-19, acute CVD is common and includes acute heart failure (3%–33%), venous thromboembolism (23%–27%), ventricular dysfunction [left ventricular (10%–41%), right ventricular (33%–47%), biventricular (3%–15%)], cardiogenic shock (9%–17%), myocardial ischemia or infarction (0.9%–11%), stress cardiomyopathy (2%–5.6%), arrhythmias (9%–17%) and arterial thrombosis secondary to virus-mediated coagulopathy [39]. Acute myocardial injury is the most described CV complication in COVID-19. Elevation of high-sensitivity cardiac troponin I (cTnI) above the upper reference limit of the 99th percentile is the most common definition [40]. Elevated cTnI levels are a marker of disease severity and underlying substrate and are associated with a greater need for mechanical ventilatory support and higher in-hospital mortality [41, 42]. Arrhythmias include arteriovenous blocks, bradycardia, and supraventricular and ventricular tachycardias [43]. Torsades de Pointes may occur due to prolongation of QT. The QT interval may be prolonged because of electrolyte changes (diarrhoea, dehydration), systemic inflammation and concomitant disease (pre-existing heart disease) [43].

Sub-clinical cardiac involvement is also extremely common in COVID-19 disease (even if often neglected). In a study of patients with mild-to-moderate COVID-19 who underwent magnetic resonance imaging of the heart, abnormal findings were noted in 78%, most commonly cardiac inflammation [44]. In other studies, cardiac inflammation and oedema were noted in 56% of the patients including in a pattern consistent with myocarditis [45, 46]. A higher incidence of cardiac complications was found with increasing age (4% in age <60 years vs 12.5% in age 60–74 years vs 31% in age ≥75 years) [47, 48].

As one would expect, myocardial injury during SARS-CoV-2 infection negatively influences patient outcome. According to a retrospective cohort study, myocardial injury was significantly associated with higher in-hospital mortality [49].

### CKD population

Apart from being at risk for severe disease, individuals with CKD also have an increased risk of CV death and CV events during COVID-19 disease. A recent, retrospective, multi-regional data-linkage study used individual patient-level data from two Scottish cohorts comprising 86.964 individuals; it suggested that CKD patients had higher rates of CV death (11.1% vs 2.7%) and CV hospitalizations (7.1% vs 3.3%) than those without CKD [50]. However, not all studies confirm the prognostic significance of CKD. In a large observational study including 8574 patients with COVID-19 from 88 US hospitals, the presence of CKD or end-stage kidney disease (ESKD) were not associated with an increased risk of death or major adverse cardiac events after multivariate adjustment [51]. Conversely, the onset of acute kidney injury (AKI) during COVID-19 disease was significantly associated with negative CV outcomes [51].

A set of more detailed studies tried to evaluate whether the risk of specific CV events observed during COVID-19 disease, such as myocardial infarction and acute coronary events, stroke, thromboembolic events, decompensated heart failure and new-onset arrhythmias is higher in patients with pre-existing CKD in comparison with patients without CKD [52]. Large retrospective studies showed higher rates of myocardial infarctions and cardiac arrests in patients with CKD, but no difference in the risk of thromboembolic events [51, 53, 54]. In the aforementioned Scottish study, higher rates of CV complications (including higher rates of fatal and nonfatal myocardial infarction, heart failure and stroke) were noted in people with CKD [50]. Again, however, these findings are not uniform. A previous retrospective cohort study of 4264 patients admitted to intensive care units with COVID-19 (12.2% with pre-existing CKD) showed that the presence of CKD was not associated with higher risk of ventricular arrhythmias or cardiac arrest or thromboembolic events [55]. With regards to individuals with ESKD on dialysis, current evidence suggests relatively high rates of CV death, as well as several complications including coronary events, new-onset heart failure and arrhythmic events during COVID-19 disease [56–59].

Overall, these data suggest that the most common acute CV complications that accompany COVID-19 disease are even more increased in patients with CKD and ESKD. However, as some of the existing studies suffer from several limitations, including retrospective nature, small sample size or relatively low event rates for specific outcomes, and lack of adequate control groups with equal burden of comorbidities, further research is necessary to shed full light onto the field.

## COVID-19 AND VENOUS THROMBOSIS: PATHOGENETIC MECHANISMS

A hypercoagulable state with widespread thrombosis and fibrinolysis has been observed in patients with severe manifestations of COVID-19 [60]. The increased coagulopathy appears to be related to a combination of underlying comorbidities, hospitalization and resulting thrombo-inflammation [61].

Endothelial cell damage following SARS-CoV-2 infection leads to disruption of physiologic anticoagulant function and development of a more procoagulant and pro-thrombotic phenotype [62]. SARS-CoV-2 destroys the endothelium and leads to cell apoptosis, which causes overexpression of coagulation factors VII, VIII and TF, and release of von Willebrand factor, activating intrinsic and extrinsic coagulation pathways [63]. The hypercoagulable state is also due to fibrinolytic abnormalities, such as increased expression of plasminogen activator inhibitor-1 (PAI-1) or downregulation of anti-thrombin, increased plasma tissue factor levels, fibrinogen, decreased ADAMTS-13 with platelet activation and inhibition of fibrinolysis [63, 64].

The hyperinflammatory state has been associated with elevated levels of markers such as interleukins, chemokines, tumour necrosis factors, interferons and various other mediators-the so-called ‘cytokine storm’ associated with disease severity [65]. COVID-19 coagulopathy has normal to borderline prolonged prothrombin time, normal or shortened aPTT, elevated D-dimers, elevated fibrinogen and normal platelet count along with microvascular thrombi [66]. Inflammatory thrombosis is associated with elevated D-dimer and acquired hypercoagulability with an increased risk of deep vein thrombosis. More rarely, immunothrombosis has been described due to heparin-induced thrombocytopenia (HIT); this is an immune complication of heparin therapy caused by pathological anti-platelet factor 4 (PF4)/heparin antibodies often causing venous or arterial thrombosis. It is possible that COVID-19 may accelerate thrombosis when this condition occurs or have a contributory role in its pathogenesis. Data on the prevalence of HIT during COVID-19 are controversial and possibly influenced by the type of assay used to test anti-PF4 antibodies, with percentages ranging between 8% [67] and 0.18% [68] of the patients admitted to ICU. It should also be considered that a large proportion of subjects undergoes prophylactic heparin therapy, thus increasing the exposure to the drugs and its complications. On the other side, multifactorial thrombocytopenia is often detected during COVID-19, making HIT probably unrecognized in many instances. HIT seems to occur more rarely in less severe forms of COVID-19 [69].

Lupus-like anticoagulant and anti-phospholipid antibodies have been also found at increased levels in COVID-19 patients [70, 71]. Their role in the hypercoagulable state of COVID-19 patients is still controversial.

## VENOUS THROMBOSIS DURING COVID-19 IN THE GENERAL POPULATION

Venous thromboembolism (VTE) [deep vein thrombosis (DVT) and pulmonary embolism (PE)] is one of the major complications of COVID-19 infection. Data on the incidence of VTE are conflicting and depend on the disease severity, the study design and the presence or not of rigorous VTE screening. In most studies, the overall incidence for VTE ranges from 7.0% to 14.1% (7.9%–22.9% for DVT and 3.5%–13.7% for PE, for non-ICU and ICU patients, respectively) [72–75]. VTE is associated with 1.37-fold increased mortality [76] and 2.5-fold increased risk of ICU admission [77].

In general, there is no benefit to prescribing anticoagulation for outpatients [78–81]. Prophylactic anticoagulation is superior to no anticoagulation in hospitalized patients and should only be given after an assessment of the bleeding risk. For specific patients with moderate to severe COVID-19, therapeutic dose anticoagulation resulted in 1.27 times more organ support–free days compared with usual thromboprophylaxis and a reduction in all-cause mortality, mechanical ventilation and ICU admission [69, 82,83]. The benefit was more evident in the high D-dimer cohort (male patients with comorbidities). For critically ill patients (ICU) the prophylactic dose was more beneficial and safer [84]. At discharge, recovered patients present a higher risk of PE and DVT (HR 3.16 and 2.55, respectively) [85], but thromboprophylaxis is recommended only for high risk patients [86, 87]. Diagnosed VTE is treated according to the general guidelines. There is no consensus on the duration of therapy (3–6 or even 12 months) [88].

Low molecular weight heparins (LMWH) are the drugs of choice due to their anticoagulant, anti-inflammatory and antiviral activities [89]. Unfractionated heparin (UFH) can also be used, but its use is limited due to strict monitoring which may expose staff to infection. Direct-acting oral anticoagulants (DOACs) use is controversial, as they do not improve clinical outcomes and increase bleeding risk, when compared with prophylactic anticoagulation [90]. Moreover, DOACs have serious interactions with antiviral drugs (especially ritonavir). Antiplatelet therapy (like aspirin) as add-on therapy in non-critically and critically ill patients is not recommended [91] as data revealed no mortality benefit and increased risk of bleeding [92].

Table 1 presents the main guidelines for thromboprophylaxis in COVID-19 patients (American Society Hematology, National Institutes of Health, International Society on Thrombosis and Haemostasis, National Institute for Health and Care Excellence) [93, 94].

**Table 1:** The main guidelines for thromboprophylaxis in COVID-19 patients from the American Society Hematology, the National Institutes of Health, the International Society on Thrombosis and Haemostasis and the National Institute for Health and Care Excellence [93, 94].

	**ASH**	**NIH**	**ISTH**	**NICE**
Update	June 2022	December 2022	July 2022	January 2023
Outpatients	No prophylaxis	No prophylaxis	Oral sulodexide for patients with high risk of disease progression	Prophylaxis, if the risk of VTE outweighs the risk of bleeding (for hospital-led acute care in the community)
Hospitalized, (non-ICU)	LMWH/UFH prophylactic dose or therapeutic dose (for patients with no organ support, D-dimers concentration >2× the upper limit)	LMWH/UFH prophylactic dose or therapeutic dose (for patients with D-dimer levels above the upper limit of normal who require conventional oxygen and who do not have an increased bleeding risk)	LMWH/UFH prophylactic dose or therapeutic dose (for patients with elevated D-dimers and increased oxygen requirements)	LMWH prophylactic dose (for patients who need low-flow oxygen and have low bleeding risk) for 7 days at least
ICU	Prophylactic dose	Prophylactic dose	Prophylactic dose	Prophylactic dose
Post-discharge	No prophylaxis	No prophylaxis	Rivaroxaban 10 mg once daily for approximately 1 month for high VTE risk patients	No recommendations

ASH, American Society Hematology; NIH, National Institutes of Health; ISTH, International Society on Thrombosis and Haemostasis; NICE, National Institute for Health and Care Excellence.

## VENOUS THROMBOSIS DURING COVID-19 IN THE CKD POPULATION

The incidence and severity of VTE are thought to be higher in the CKD population, due to comorbidities and hypercoagulation status. However, in a recent meta-analysis, renal disease was not a risk factor for VTE [95]. On the other hand, in patients with diagnosed VTE, CKD is associated with increased mortality, VTE recurrence and major bleeding [96].

As far as we know, there are no studies investigating certain anticoagulant strategies in COVID-19 patients with CKD, and CKD patients are poorly represented in randomized controlled trials (e.g. 7%–11%) [95, 96]. The main challenges for clinicians are the assessment of bleeding and thrombosis risk (stratification tools are not validated in CKD populations), optimal duration, type and dose of anticoagulation therapy. Moreover, D-dimers levels, which are associated with severity, mortality and VTE in COVID-19 patients [97], are dependent on eGFR in CKD patients. Although high levels still predict VTE, cut-off values should be adjusted (especially for PE) [98, 99].

Data from the general population and common sense justify the use of standard prophylactic doses of LMWH (e.g. enoxaparin 40 mg subcutaneously daily) or UFH (5000 IU subcutaneously twice daily) in the CKD population. In hospitalized CKD patients who meet the criteria for therapeutic dose as described above, higher doses of LMWH (e.g. enoxaparin 1 mg/kg twice daily) may be chosen. For eGFR 15–29 mL/min/1.73 m^2^, renal dose adjustment of LMWH should be considered for either prophylaxis (e.g. enoxaparin 30 mg subcutaneously daily) or treatment (e.g. enoxaparin 1 mg/kg subcutaneously daily). For patients with eGFR <15 mL/min, UFH is ideal either for prophylaxis or treatment (80 IU/kg bolus intravenously followed by 18 IU/kg/h infusion, under close monitoring). Fondaparinux should be avoided especially in cases of unstable renal function or with an eGFR <30 mL/min [100].

Regarding anticoagulation in CKD patients with COVID-19, meticulous follow-up, dose adjustments according to eGFR and individualization of the therapy in relation to the patient comorbidities, history and risk factors for bleeding or thrombosis are the best approach and management to date [101].

The hypercoagulability state affecting COVID-19 patients appears to be also associated with increased filter and lines clotting during continuous renal replacement therapy (CRRT) or intermittent haemodialysis. In this respect, a multicentre study of consecutive patients with COVID-19 receiving CRRT showed that 83% of the patients lost at least one filter during CRRT [102]. Reinforcement of the anticoagulation strategy by adding heparin to a standard regional anticoagulation with citrate has been proposed [103].

## LONG COVID AND IMPLICATION FOR CARDIOVASCULAR DISEASE

The World Health Organisation (WHO) has defined the ‘post COVID-19 condition’, more widely termed ‘long COVID’, as continuation or development of new symptoms 3 months after initial SARS-CoV-2 infection, with these symptoms lasting for at least 2 months with no other explanation. Symptoms may include fatigue, malaise, dyspnoea, cough, chest pain, palpitations, arthralgia, poor memory, cognitive dysfunction and rash, amongst others. A large US electronic health records cohort study suggests that there are six phenotypic subtypes of long COVID, depending on the symptom cluster [104]. Long-COVID patients with persistent elevation in serum creatinine (suggesting either pre-existing CKD, *de novo* CKD and/or non-resolution of AKI) falls within a cluster characterized by more severe clinical disease, with other laboratory abnormalities and a higher mortality [104].

AKI is common in patients hospitalized with COVID-19 infection and is associated with increased risk of short-term mortality in multiple cohort studies [105, 106]. In long-term cohort studies of patients surviving hospitalization with COVID-19, increased severity of AKI is associated with worse long-term kidney function over long-term follow-up [107]. In another US Veterans cohort study, both hospitalized and non-hospitalized survivors of COVID-19 infection have more rapid decline in eGFR (95% CI in parentheses) over median follow up of 164 days compared with non-infected controls [–3.26 (–3.58 to –2.94) and –5.20 (–6.24 to –4.16) mL/min/1.73 m^2^/year in non-hospitalized and hospitalized patients, respectively] [108], although this study was largely in people infected in the pre-vaccination era.

Therefore, COVID-19 infection is associated with a long-term decline in kidney function, which is a component of long-COVID syndromes. Declines in eGFR post-COVID-19 infection are exacerbated by severity of acute COVID-19 infection and pre-existing CKD. It is widely established that reduction in eGFR is a risk factor for future CVD, and that presence of CKD and/or eGFR decrease should be applied to prognostic CV risk calculators [109, 110].

It is unknown to what degree reduction in eGFR associated with COVID-19 infection may indirectly impact on increased CV risk. The risk of multiple incident CVD subtypes has been shown to be elevated in the year following COVID-19 infection [111]. Furthermore, CV risk is increased after AKI more widely [112] so there may be an indirect impact of AKI and subsequent loss in GFR on CV risk in addition to any specific CV impact of COVID-19.

Apart from AKI, a substantial percentage of patients develops proteinuria during SARS-CoV-2 infection [113]. There are several potential reasons for this, some due to a possible direct pathogenetic effect of the virus in the kidney and others due to the systemic effects of the infection and critical illness. Unfortunately, this aspect has been little investigated during the pandemic. In addition, little information is available on proteinuria in patients developing long COVID and its implication on the amplification of CV risk.

The prolonged inflammatory and procoagulant state of long COVID may have implications from the CV point of view. According to an epidemiological study performed in England, among 47 780 subjects with COVID-19 and discharged alive before vaccine availability, major adverse CV events (MACE) were diagnosed three times more frequently than in a matched cohort from the general population [114]. Similar findings were reported from a large database of the US Department of Veterans Affairs [115]. Compared with a contemporary control cohort, at 12 months those with COVID-19 had an increased risk of stroke (HR 1.52, 95% CI 1.43–1.62), atrial fibrillation (HR 1.71, 95% CI 1.64–1.79), ischaemic heart disease outcomes (HR 1.66, 95% CI 1.52–1.80) and heart failure (HR 1.72, CI 1.65–1.80) [115]. An increased risk for thromboembolic disorders was also described [115]. Recently, a systemic review collected information from seven studies including 1 321 305 post-COVID-19 cases [116]; despite similar survival rates to control cohorts, post-COVID-19 individuals were confirmed having a higher risk of MACE [115].

Little information is available for the CKD population regarding the incidence of long COVID and its CV effects. In any case, data from small cohorts of haemodialysis patients suggest increased mortality and adverse events in the months following a SARS-CoV-2 infection [117, 118].

## THE ROLE OF VACCINATION AND DIFFERENT SARS-CoV-2 VARIANTS

Currently available vaccines include those based on genetically recombinant mRNA molecules (BNT 162b2 Pfizer/BioNTech, mRNA-1273 Moderna) and non-replicating viral vector vaccines (CgAdOx1nCoV-19 AstraZeneca/Oxford, Ad26.COV2.S Janssen/Johnson & Johnson and Sputnik V Gamaleya Research). Vaccines are safe even in high-risk populations and have dramatically reduced the risk of severe COVID-19 [119].

Little is known about CVD after vaccination; nevertheless, results from the US National COVID Cohort Collaborative (N3C) in patients immunized with mRNA and viral vector vaccines showed that both full and partial vaccination were associated with a lower risk of MACE 2 weeks after vaccination in a diverse population of race, age and health records [120]. These results were consistent with those of smaller cohorts showing a reduced risk of MI or stroke within 14 days after COVID-19 vaccination [121]. However, adverse effects have been reported in smaller number of patients, particularly in the elderly or those with a pre-existing high risk, such as hypertension, platelet aggregation, thrombosis, arrhythmia, myocarditis, acute kidney injury (AKI) with proteinuria, haematuria secondary to podocyte injury, neurological or psychiatric disorders [119, 122].

Vaccines may be less effective in subpopulations of patients with CKD, especially those on dialysis or under immunosuppressive treatment, in terms anti-spike antibody levels, hospitalization and mortality [123, 124]. In patients receiving renal replacement therapy (including kidney recipients), mRNA-1273 induced higher antibody levels, but also increased incidence of adverse events, mostly at the site of vaccination, while CV complications were not reported in many studies, regardless of the type of vaccine used [125, 126]. The administration of a third booster dose had a significant effect on antibody levels, even in patients with a lower initial response, and reduced disease severity and morbidity [127, 128], and a fourth dose helped to increase anti-spike antibody titres [129]. Unfortunately, patients with higher vaccine requirements (those with lower antibody levels or kidney transplant recipients) had the lower antibody titres after the booster dose. The third or fourth dose were not associated with higher CV complications in the vaccinated CKD population [128].

## POSSIBLE TREATMENTS AND PREVENTION STRATEGIES

Since the start of the pandemic over 3 years ago, several effective treatments have become available. This is the source of continuous, almost frenzied, research activity with newer agents coming forward and emerging new variants of the virus. Over 5000 trials registered or are ongoing [130, 131]. Although most of these studies are small and of variable methodological quality, some large, international platform trials have provided robust evidence. Such trials can also adapt their design, recruitment strategies and selection of interventions based on new insights. Examples include ACCT, RECOVERY, WHO SOLIDARITY, REMAP-CAP and ACTIV, which recruit large numbers of patients in many countries [132–135]. An overview of ongoing trials is available from the Infectious Diseases Data Observatory through their living systematic review of COVID-19 clinical trial registrations [136] and the World Health Organization (WHO) website (https://www.covid-nma.com/dataviz/).

To deal with the rapid developments and worldwide need for up-to-date information the WHO has created a living guideline to deal with the evidence and dynamically update it [136]. This guideline is linked to living network meta-analyses [137, 138] and other regularly updated resources [136]. The current recommendations (updated on 13 January 2023) are summarized in Table 2.

**Table 2:** Recommended treatments for patients confirmed with COVID-19 (adapted from WHO Living Guideline 13 January 2023).

	Population		
	Non-severe	Severe	Critical
	Absence of signs of severe or critical disease	Oxygen saturation <90% on room air, signs of pneumonia, signs of severe respiratory distress (use of accessory muscles, unable to speak in full sentences, respiratory rate >30/min)	Requires life support, acute respiratory distress syndrome, sepsis, septic shock
Interventions			
Strong recommendations in favour	Nirmatrelvir and ritonavir^a^	Corticosteroids, IL-6 receptor blockers, baricinib	
Weak or conditional recommendations in favour	Molnupiravir^a^, remdesivir^d^	Remdesivir^d^	
Weak or conditional recommendations against	Corticosteroids, invermectin^c^, fluvoxamine^c^	Ruxolitinib and tofacitinib^b^, convalescent plasma^c^	Remdesivir^d^
Strong recommendations against	Convalescent plasma, colchicine, hydroxychloroquine, lopinavir–ritonavir, casirivimab and imdevimab, sotrovimab		

a For those at >10% risk of hospital admission. Typically, it will include unvaccinated people, older people, immunodeficiency and chronic diseases.

b Should be considered only if neither baricinib nor IL-6 receptor blockers are available.

c Only in research settings.

d Contraindicated for eGFR <30 mL/min 1.73 m^2^. However, the drug has been used safely also in patients with lower GFR [142, 143].

It should be noted that these guidelines and resources do not necessarily make renal specific recommendations. Clinicians should always check for any dose alterations that might be required for renal function as well as potential drug interactions with specific publications [139, 140], as well as national and regional guidelines [141].

## SUMMARY

CVD is now recognized as a significant complication of SARS-CoV-2 infection. Its pathogenesis is multifactorial and still not completely understood. While a direct viral cytopathic effect remains controversial, the combination of accelerated inflammation together with a hypercoagulable state are the most likely triggers.

CKD patients are at high risk for CV complications during SARS-CoV-2 infection for multiple reasons: they often develop severe forms of COVID-19, CKD is an inflammatory condition *per se* making CKD an important CV risk factor, and because CKD patients have other comorbidities further enhancing their CV risk. The awareness of chronic implications of COVID-19 on CVD is still limited, especially for the CKD population.

## Data Availability

No new data were generated or analysed in support of this research.
